# Brain activity during cybersickness: a scoping review

**DOI:** 10.1007/s10055-023-00795-y

**Published:** 2023-04-12

**Authors:** Eunhee Chang, Mark Billinghurst, Byounghyun Yoo

**Affiliations:** 1 Empathic Computing Laboratory, University of South Australia, Mawson Lakes, SA Australia; 2 Center for Artificial Intelligence, Korea Institute of Science and Technology, 5 Hwarangro14-gil Seongbuk-gu, Seoul, 02792 South Korea; 3 Artificial Intelligence and Robotics, KIST School, Korea University of Science and Technology, 5 Hwarangro14-gil, Seongbuk-gu, Seoul, 02792 South Korea

**Keywords:** Cybersickness, VR sickness, Electroencephalogram, Virtual reality

## Abstract

Virtual reality (VR) experiences can cause a range of negative symptoms such as nausea, disorientation, and oculomotor discomfort, which is collectively called cybersickness. Previous studies have attempted to develop a reliable measure for detecting cybersickness instead of using questionnaires, and electroencephalogram (EEG) has been regarded as one of the possible alternatives. However, despite the increasing interest, little is known about which brain activities are consistently associated with cybersickness and what types of methods should be adopted for measuring discomfort through brain activity. We conducted a scoping review of 33 experimental studies in cybersickness and EEG found through database searches and screening. To understand these studies, we organized the pipeline of EEG analysis into four steps (preprocessing, feature extraction, feature selection, classification) and surveyed the characteristics of each step. The results showed that most studies performed frequency or time-frequency analysis for EEG feature extraction. A part of the studies applied a classification model to predict cybersickness indicating an accuracy between 79 and 100%. These studies tended to use HMD-based VR with a portable EEG headset for measuring brain activity. Most VR content shown was scenic views such as driving or navigating a road, and the age of participants was limited to people in their 20 s. This scoping review contributes to presenting an overview of cybersickness-related EEG research and establishing directions for future work.

**Supplementary Information:**

The online version contains supplementary material available at 10.1007/s10055-023-00795-y.

## Introduction

Virtual reality (VR) experiences can cause uncomfortable body states called cybersickness or VR sickness. Depending on the experimental setup, 60–80% of users reported some symptoms of cybersickness (Ahn et al. 2020; Kim et al. 2005), and the discomfort can last an hour or up to 5 h after the VR experience (Rebenitsch and Owen 2016; Regan and Price 1994). Three common symptoms are frequently observed during cybersickness; nausea, disorientation, and oculomotor discomfort (Kennedy et al. 1993). To understand and manage these negative symptoms, there is a need to accurately quantify the severity of cybersickness.

This is often done by using a questionnaire, such as the simulator sickness questionnaire (SSQ) (Kennedy et al. 1993), fast motion sickness scale (FMS) (Keshavarz and Hecht 2011), or misery scale (Bos et al. 2010). The SSQ is the most widely used questionnaire for measuring the subjective level of cybersickness. The questionnaire contains sixteen symptoms, and participants can report the severity of their discomfort by choosing a number (0: no symptom; 1: mild; 2: moderate; 3: severe) on a rating item. The FMS is rather simpler, allowing participants to verbally report a number between 0 (no sickness at all) and 20 (frank sickness). These methods are easy to adopt and provide an intuitive way of measuring the subjective level of cybersickness.

However, these approaches depend on a person’s subjective judgment, so it is hard to generalize among participants. Moreover, it is challenging to instantly measure the level of cybersickness during the VR experience. Therefore, there has been an effort to adopt a more objective and on-time method for quantifying the discomfort. Several physiological signals such as an electrocardiogram (ECG), electrooculogram (EOG), electrogastrogram (EGG), respiration (RSP), and skin conductivity have been suggested for promising alternatives (Davis et al. 2014). While participants experience VR, their physiological responses can be recorded simultaneously to monitor and detect uncomfortable body states. Previous research has shown significant changes in heart rate, blink rate, and stomach activity when participants felt discomfort (Dennison et al. 2019; Kim et al. 2005). Garcia-Agundez et al. (2019) developed a cybersickness classification model based on a combination of biosignals, showing a maximum accuracy of 82%.

Brain activity monitoring using electroencephalogram (EEG) sensing is also one technique for obtaining an objective measure (Chang et al. 2020). While the autonomic nervous system variables (e.g., ECG, RSP, skin conductivity, etc.) might take several seconds to respond to the stressful event, the EEG signal can instantly (milliseconds time scale) reflect the bodily changes due to the pain (Shao et al. 2011). Using the EEG’s high temporal resolution, previous studies have investigated which brain activities are highly engaged in transient neural changes during cybersickness (Chang et al. 2022; Chen et al. 2010; Kim et al. 2005). Moreover, the EEG signal can provide both temporal and spatial aspects of neural processing; therefore, this approach can elucidate which brain areas are related to the onset of the negative symptom. Based on these characteristics, several researchers have focused on extracting and selecting specific EEG features for detecting and measuring discomfort using various up-to-date algorithms (Dennison et al. 2019; Li et al. 2019).

Previous research by Kim et al. (2008) showed great insight into cybersickness-related EEG research in the late 2000s (Kim et al. 2008). The authors implemented a 9-channel EEG system and developed a cybersickness relief virtual environment (CRVE) based on an artificial neural network. Several promising EEG features, selected through a principal component analysis (PCA), were used to predict the subjective level of cybersickness. The authors let the system manipulate the field of view (FOV) of the VR screen according to the prediction made by the CRVE system (e.g., reducing the FOV when the CRVE judges a participant is experiencing cybersickness). The results showed that this system can significantly reduce discomfort compared to the random FOV manipulation system (i.e., non-CRVE). This earlier work contributed to providing experimental evidence that the user’s brain waves can serve as a predictor of cybersickness and also as a controller of a VR system to reduce cybersickness.

Progress in machine learning and deep learning research provides new insights for detecting cybersickness (Matsushita et al. 2021). Considering the subjective report from participants as the ground truth, several proposed models tried to accurately detect users’ discomfort using EEG features such as theta or alpha band power (Kim et al. 2008; Lee et al. 2021). Most studies have extracted specific neural correlates to predict the subjective cybersickness score. Recent studies also consider content features (e.g., exceptional movement, acceleration) or other physiological signals (e.g., ECG, EGG, RSP, body sway) to improve the accuracy of detection (Dennison et al. 2019; Garcia-Agundez et al. 2019; Kim et al. 2019a).

**Table 1:** Summary of earlier related surveys including EEG research in VR

Reference	Review scope	Considered venues	Coverage pipelines for EEG analysis	Coverage years	# of articles (EEG &CS/total)
Yildirim (2020)	EEG-based cybersickness classification	Web of science, PubMed, Google scholar	Feature extraction Classification	$\sim$ 2020*	4/4
Wang and Suh (2021)	EEG approach in VR/AR/MR	Scopus	Feature extraction	$\sim$ 2017*	3/84
Halbig and Latoschik (2021)	Physiological measurements in VR	ACM digital library, Web of science, PubMed, APA PsycInfo, PsynDex, IEEE xplore	Classification	$\sim$ 2020	1/32
This survey	EEG research in cybersickness	Web of Science, Google Scholar	Preprocessing Feature extraction Feature selection Classification	$\sim$ 2021	33/33

*The coverage years is not explicitly reported in the paper but can be assumed based on the results of selected papers

Despite the increasing interest, little is known about whether there is a consistency in neural correlates of cybersickness and what kinds of equipment and analysis have been adopted for identifying the cybersickness-related EEG marker. For these reasons, we conducted a scoping review to systematically organize the previous research on changes in brain activity induced by cybersickness. Based on the guidelines of Preferred Reporting Items for Systematic reviews and Meta-Analyses extension for Scoping Reviews (PRISMA-ScR) (Tricco et al. 2018), we reviewed original research papers that investigated EEG for measuring and predicting cybersickness. In this survey, we aim to answer the following research questions:

1. Are there any consistent results indicating a specific EEG marker for cybersickness that can reliably detect the negative symptom?
2. How accurate is the EEG classification model for detecting cybersickness?
3. Which experimental setups and designs were being used in cybersickness-related EEG research?

The main contribution of this paper is providing a systematic review of EEG-based cybersickness research. Though the previous surveys focused on EEG approaches in various VR contexts, only a limited number of studies were selected for reviewing the neural correlates of cybersickness. Moreover, most previous surveys focused only on a part of the EEG analysis pipeline such as feature extraction or classification. In the present study, we categorized the entire EEG analysis into four steps (preprocessing, feature extraction, feature selection, and classification) and covered all steps of EEG analysis. Then, we investigated the hardware, content, and other experimental setups of the selected studies.

## Related work

Several related surveys reviewed EEG approaches in VR research (Table 1). Yildirim (2020) focused on the increasing demand for detecting cybersickness based on deep learning algorithms. Based on the eligibility criteria, the author selected four previous studies which adopted deep learning frameworks to detect the subjective level of discomfort. This review reports an average classification accuracy of each study and investigated which algorithms were adopted for the classification. However, as the author pointed out, this survey covered a limited number of previous studies in the review.

Wang and Suh (2021) also emphasized the significance of the EEG approach in immersive technology including augmented reality, virtual reality, and mixed reality. Instead of focusing on cybersickness-related brain activity, their survey reviewed a broader range of users’ states such as mental load, embodiment, postural control, etc. In particular, the author regarded cybersickness as an abnormal state of postural control, and briefly mentioned the possibility of alpha and gamma power engagement in experiencing cybersickness.

A recent study by Halbig and Latoschik (2021) conducted a comprehensive survey on physiological measurements and VR applications. The authors of the study reviewed 1,119 previous works and identified 32 papers, which investigated the classification of common experiences in VR (e.g., cognitive workload, stress, anxiety, etc.) using physiological approaches. Their review thoroughly reported the sensor information, sample size, content information, and details of classification methods of the selected studies. Among the selected studies, however, only one study focused on brain activity under cybersickness (Jeong et al. 2019).

In the present survey, we reviewed 33 original research papers on EEG-based cybersickness. Among many physiological approaches, we specifically targeted brain activity during cybersickness and investigated whether EEG can be used to detect users’ discomfort using up-to-date machine learning or deep learning algorithm. Moreover, we systematically reviewed the entire pipeline of previous EEG-cybersickness research. Based on review of our four steps of the EEG analysis pipeline, we report overall experimental methods from data acquisition to the classification model.

**Fig. 1:**
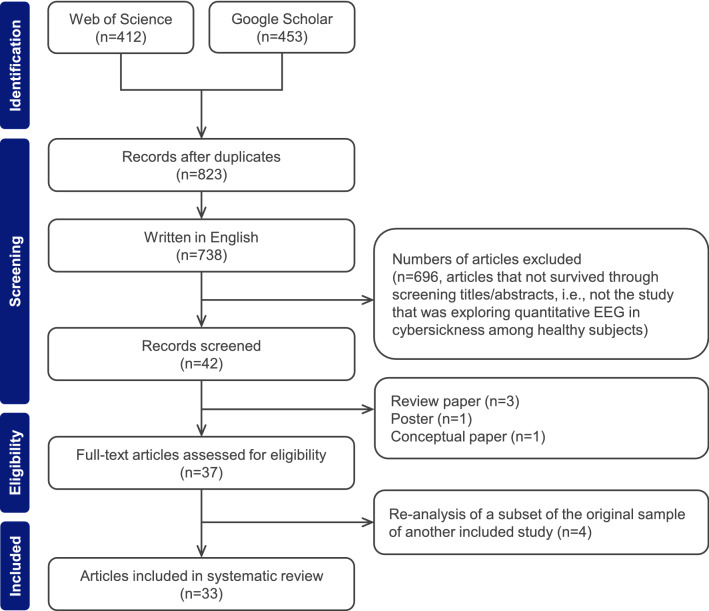
PRISMA flowchart for the study selection

## Method

### Data sources and search strategy

We conducted this review following the PRISMA-ScR guidelines (http://www.prisma-statement.org/Extensions/ScopingReviews) (Supplementary Materials, Appendix 1). We searched articles on both the Web of Science and Google Scholar, and the date of search was September 20th, 2021. The search keywords were outlined by two authors (EC and BY) and further refined through team discussion. Finally, we selected the terms ‘cybersickness’, ‘VR sickness’, ‘electroencephalogram’, and ‘EEG’ as inclusion keywords and ‘therapy’ as an exclusion keyword. The combination of the search terms was as follows: {"electroencephalogram" OR “EEG”} AND {“cybersickness”} AND {“VR sickness”} NOT {"therapy"}. Note that the terms “cybersickness” and “VR sickness” in this paper indicate not only visually induced motion sickness but also the user’s discomfort caused by a motion bed (Chen et al. 2010; Ko et al. 2013; Lin et al. 2007; Liu et al. 2020; Wei et al. 2011).

### Eligibility criteria

We set five eligibility criteria for our scoping review based on the search strategy.

1. Publications before 2021–09-20 (including Early Access)
2. Publications written in English
3. Publications providing quantitative results using EEG analysis (e.g., statistical results, accuracy, etc.)
4. Publications aimed at healthy participants (i.e., no history of brain injury or visual/vestibular malfunction)
5. Original Research publications

We excluded cybersickness research during VR therapy which was aimed at patients (e.g., abnormal vestibular function and/or mental diseases). We also did not consider a review, poster, or proof-of-concept paper that did not contain any experimental results. Finally, we excluded four articles that re-analyzed a subset of the original research.

**Fig. 2:**
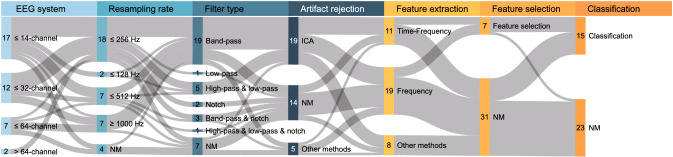
Sankey diagram of EEG analysis pipeline

### Data extraction

Two authors (EC and BY) developed a data-charting form and determined which variables would be included for further analysis. After charting the data, the authors discussed the results and iteratively updated the form. Based on this process, we extracted data items in four categories from the EEG analysis pipelines: preprocessing, feature extraction, feature selection, and classification. Preprocessing was applied to all EEG experiments to acquire noise-reduced brain activity, and we investigated each preprocessing step in detail. After that, depending on their research purpose, previous studies conducted any of three steps of EEG analysis (i.e., feature extraction, feature selection, and classification). We described which EEG feature extraction (e.g., frequency analysis, time-frequency analysis, and other methods), selection algorithm (e.g., principal component analysis (PCA), genetic algorithm (GA)), or classification model (e.g., support vector machine (SVM), convolutional neural network (CNN), deep neural network (DNN)) was frequently used in the research.

Finally, we also covered hardware factors (e.g., types of the VR display, EEG configuration), content factors (e.g., type of VR content, duration, baseline content), and other experimental factors (e.g., sample size, average age, subjective measures for cybersickness) to find any common elements between the studies.

## Results

From the initial search, we had 412 Web of Science articles and 453 Google Scholar articles. We eliminated duplicate records and then selected articles by screening titles and abstracts depending on the inclusion and exclusion criteria. As mentioned above, we only considered original research providing quantitative experimental results. Applying the screening, eligibility and inclusion criteria finally resulted in 33 full-text publications (Fig. 1).

Among the 33 articles, Ahn et al. (2020) adopted three different analyses in one study for the feature extraction. Kim et al. (2019b), Liu et al. (2020), and Wei et al. (2019) performed two distinguished approaches in their studies, respectively (Supplementary Materials, Appendix 2). Therefore, 38 pieces of research were selected in total if we counted those analyses as separate research items. We surveyed 33 articles for the preprocessing (Sect. 4.1.1) and experimental setups (Sects. 4.2, 4.3, 4.4) because each article adopted the same procedure and material regardless of the types of analysis performed. However, 38 studies were reviewed for the feature extraction (Sect. 4.1.2), feature selection (Sect. 4.1.3), and classification (Sect. 4.1.4) to thoroughly survey the approaches used.

Figure 2 shows a Sankey diagram indicating an entire pipeline of cybersickness-related EEG research (33 selected papers, but 38 experiments in total to illustrate all pipelines at once). We visualize previous research trends from the experimental configuration to EEG classification using this diagram. The seven node types represent the EEG system (number of channels), resampling rate, filter type, artifact rejection methods, feature extraction methods, feature selection, and classification, respectively. The number on each node refers to the number of experiments with this property (e.g., the number of experiments using more than 64 channel EEG systems). In the next section, we focus on the EEG analysis procedure, which consists of four parts: preprocessing, feature extraction, feature selection, and classification. After that, we also cover the hardware factors, content factors, and other experimental factors of the selected studies.

**Fig. 3:**
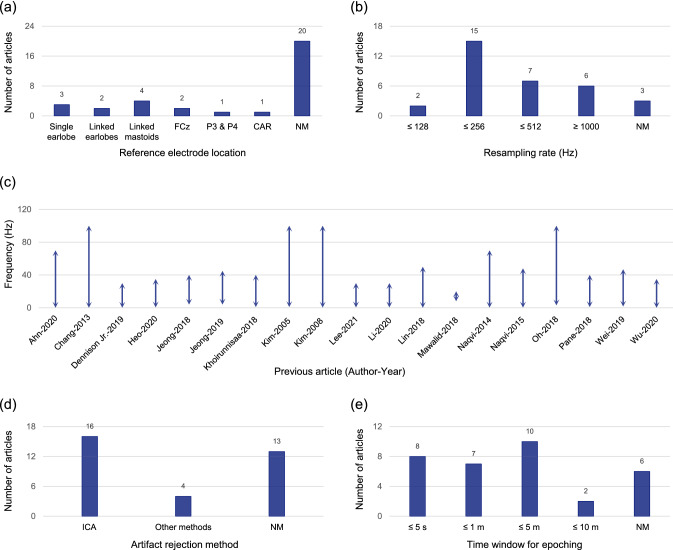
Distributions of suggested approaches in each preprocessing step** a** re-referencing location,** b** resampling rate,** c** cut-off frequency range of the band-pass filter,** d** suggested artifact rejection method, and** e** epoch size

### EEG analysis pipeline

#### Preprocessing

Regardless of the types of data analysis, a participant’s raw EEG data should be preprocessed for better signal-to-noise ratio. In most cases, the preprocessing procedure is standardized into the following steps; re-reference, resampling, filtering, artifact rejection, and epoching. We investigated the preprocessing details of the selected 33 research papers and described the trends in each step (Table 2).

**Table 2:** Summary of the preprocessing details for included studies

References	Reference electrode loc.	Resampling rate (Hz)	Filter (cut-off frequency)	Artifact rejection	Epoch
Ahn et al. (2020)	FCz	512 or 256	Band-pass filter (0.1–70 Hz)	ICA	$\le$ 1 min
Celikcan (2019)	CAR	NM	NM	ICA	NM
Chang et al. (2013)	Linked earlobes	1000	Band-pass filter (0.1–100 Hz)	ICA	$\le$ 1 min
Chen et al. (2010)	NM	250	High-pass (1 Hz)	ICA	$\le$ 5 min
			Low-pass (50 Hz)		
Choi et al. (2009)	Single earlobe	256	Low-pass filter (30Hz)	NM	$\le$ 5 min
Dennison et al. (2019)	NM	1024	Band-pass filter (0.1–30 Hz)	ICA	$\le$ 1 min
Heo and Yoon (2020)	Linked mastoids	1000	Band-pass filter (0.5–35 Hz)	ICA	NM
Jeong et al. (2018)	NM	NM	Band-pass filter (4–40 Hz)	NM	NM
Jeong et al. (2019)	NM	128	Band-pass filter (4–45 Hz)	Other methods	NM
Khoirunnisaa et al. (2018)	P3 and P4	256	Band-pass filter (1–40 Hz)	ICA	$\le$ 5 min
Kim et al. (2005)	Single earlobe	400	Band-pass filter (1–100 Hz)	NM	$\le$ 1 min
			Notch filter (60 Hz)		
Kim et al. (2008)	Single earlobe	400	High-pass filter (1 Hz)	NM	$\le$ 1 min
			Low-pass filter (100 Hz)		
			Notch filter (60 Hz)		
Kim et al. (2019a)	NM	250	Band-pass filter (0.3–100 Hz)	NM	NM
			Notch filter (60 Hz)		
Kim et al. (2019b)	NM	NM	NM	Other methods	NM
Ko et al. (2013)	NM	250	High-pass filter (1 Hz)	ICA	$\le$ 5 min
			Low-pass filter (50 Hz)		
Krokos and Varshney (2022)	NM	128	High-pass filter (1 Hz)	ICA	$\le$ 5 min
			Low-pass filter (50 Hz)		
Lee et al. (2019a)	NM	1000	NM	NM	NM
Lee and Alamaniotis (2020)	NM	256	NM	NM	$\le$ 5 s
Lee et al. (2021)	Linked earlobes	1000	Band-pass filter (0.5–30 Hz)	NM	$\le$ 5 min
Li et al. (2019)	Linked mastoids	200	NM	NM	$\le$ 5 s
Li et al. (2020)	NM	256	Band-pass filter (0.5–30 Hz)	NM	$\le$ 5 s
Liao et al. (2020)	NM	512	NM	NM	$\le$ 10 min
Lin et al. (2007)	NM	500	High-pass filter (1 Hz)	ICA	$\le$ 5 min
			Low-pass filter (50 Hz)		
Lin et al. (2018)	Linked mastoids	500	Band-pass filter (0.1–50 Hz)	ICA	$\le$ 10 min
Liu et al. (2020)	NM	220	Notch filter (NM)	NM	$\le$ 5 min
Mawalid et al. (2018)	NM	256	Band-pass filter (8–20 Hz)	ICA	$\le$ 5 min
Naqvi et al. (2014)	NM	250	Band-pass filter (0.3–70Hz)	Other methods	NM
			Notch filter (50 Hz)		
Naqvi et al. (2015)	NM	250	Band-pass filter (0.3–48Hz)	Other methods	$\le$ 1 min
Oh and Whangbo (2018)	NM	512	Band-pass filter (3–100Hz)	NM	NM
Pane et al. (2018)	NM	256	Band-pass filter (1–40 Hz)	ICA	$\le$ 5 min
Wei et al. (2011)	NM	250	High-pass filter (1 Hz)	ICA	$\le$ 5 min
			Low-pass filter (50 Hz)		
Wei et al. (2019)	Linked mastoids	1000	Band-pass filter (1.6–47 Hz)	ICA	$\le$ 5 s
Wu et al. (2020)	FCz	500	Band-pass filter (0.1–35 Hz)	ICA	$\le$ 5 s

*CAR* common average reference, *NM* not mentioned, *ICA* independent component analysis

From this table, about 40% of the previous studies (i.e., 13/33) indicated which electrodes were used for the re-reference (Fig. 3a). The signal at a particular electrode, the average of two mastoids or earlobes, or the overall signal average (i.e., common average reference (CAR)) is commonly chosen for a reference signal (Bigdely-Shamlo et al. 2015). According to the survey, the earlobes or mastoids were most often selected as reference electrodes. These locations are well-known places for the reference electrode because they are electrically stabilized but linked to the cortical area. In some cases, a particular spherical region, such as the frontal or parietal area, was chosen for the re-referencing (Ahn et al. 2020; Khoirunnisaa et al. 2018; Wu et al. 2020).

The signals from an EEG amplifier are usually down-sampled for further analysis. Only three papers did not indicate both the sampling rate of the device and the resampling rate (Celikcan 2019; Jeong et al. 2018; Kim et al. 2019b). Except for those papers, we organized the resampling rate of the previous works and illustrated the trend in Fig. 3b. In case of the article did not indicate the resampling information, we regarded the resampling rate and the device sampling rate as the same. The result showed that most studies resampled the raw data at between 128 and 256 Hz. This rate covers the typical frequency band for EEG analysis (< 50 Hz) and successfully satisfies Nyquist’s theorem for signal processing.

The resampled EEG data can be filtered to remove noise. The survey showed that most studies applied a band-pass filter (Table 2, 4th column). Otherwise, the study of Choi et al. (2009) adopted a low pass filter (cut-off frequency: 30 Hz), and Kim et al. (2005, 2008) applied an additional notch filter to eliminate the 60 Hz line noise. Figure 3c indicates a distribution of the cut-off frequency of the band-pass filter in each study. In most cases, filters were designed to pass the 0.1–60 Hz brain signal so that the spectral power of the target frequency (< 50 Hz) remained largely unaffected.

Electrooculogram (EOG) is one of the major artifacts in brain signals. Eyeball movements can induce electric dipoles and disrupt the EEG quality. Generally, an independent component analysis (ICA) is widely applied to remove various types of artifacts (e.g., muscle components, electrocardiogram, bad channels, etc.) including EOG. According to our survey, about half of the selected articles reported their method of removing EOG (16/33) (Fig. 3d). Most studies applied ICA or ICA-based approaches (e.g., second-order blind identification, ADJUST toolbox). Other studies adopted a built-in algorithm in the EEG device (Naqvi et al. 2015) or a visual inspection (Kim et al. 2019b).

After the artifact rejection, continuous EEG data were epoched for feature extraction. Figure 3e shows a distribution of the time window for each study. For the event-related potential (ERP) or binary label analysis, the duration of the time window was less than 10 s. On the other hand, the authors of prior work set a target window between 1 and 5 min for the frequency or time-frequency analysis. The longest epoch was 10 min in the study of (Liao et al. 2020; Lin et al. 2018).

**Fig. 4:**
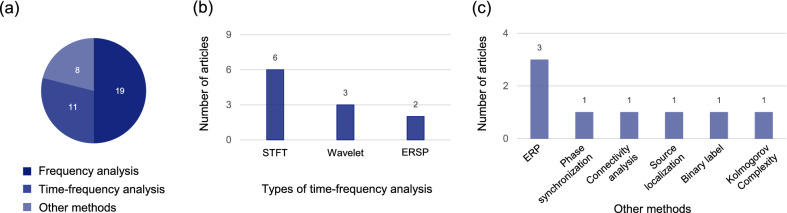
Distributions of suggested approaches in** a** feature extraction,** b** time-frequency analysis, and** c** other methods. *ERP* event-related potential,* ERSP* event-related spectral perturbation,* STFT* short-time Fourier transform

From the surveyed papers participants watched VR content for 3 s at least (Wei et al. 2019) to 60 min at most (Choi et al. 2009; Lee et al. 2021) (see more details in subsection 4.4), and a limited portion of the signal was used for the analysis. For example, Kim et al. (2005) and Chang et al. (2013) divided the early, mid, and later parts of the VR experience and compared the neural activity between them. This approach was based on the well-known fact that cybersickness worsens as the length of time spent experiencing content increases (Chang et al. 2020). Alternatively, the level of discomfort was continuously recorded using a joystick, and then a section where the response was severe was extracted for the analysis (Chen et al. 2010; Ko et al. 2013; Wei et al. 2011).

#### Feature extraction

After preprocessing, cybersickness-related EEG features were extracted depending on the research purposes. Many different types of approaches have been performed to investigate the neural correlates of cybersickness. As mentioned earlier, we investigated which methods of feature extraction were used for the total 38 pieces of research (Table 3). We also surveyed the target frequency, major results, and whether the feature selection or classification was performed or not.

The results showed that previous research performed various analyses such as using fast Fourier transform (FFT), short-time Fourier transform (STFT), and event-related potential (ERP). We categorized these approaches into three parts depending on the domain of EEG analysis: (1) frequency analysis, (2) time-frequency analysis, and (3) other methods. Figure 4 describes the portion of each approach in the feature extraction (a) and their detailed distributions (b and c).

Frequency analysis derives the power of a specific frequency band by applying FFT to time series brain waves. Among the 38 cases, 19 experiments performed FFT. Previous studies investigated the changes in power below 50 Hz, which consists of five frequency bands; delta, theta, alpha, beta, and gamma. Most studies presented a specific frequency range for each band or mentioned only the name of the target band (Table 3, 3rd column). The relative power of each band was also often computed rather than the absolute power (Table 3, 4th column).

**Fig. 5:**
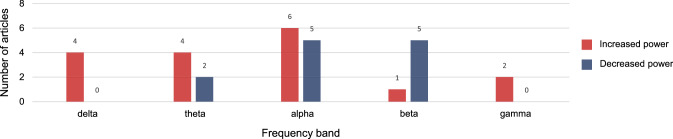
Spectral power changes of each frequency band in a higher level of cybersickness

Time-frequency analysis was also widely used for feature extraction. Since the frequency analysis loses the EEG temporal information, many studies have tried to adopt a time-frequency approach to overcome this limitation. For example, while the FFT derives a single power level of the target frequency band for the entire time range (e.g., beta power (dB) for 1 min), the time-frequency analysis can provide the power changes during the time range by adopting the moving window of FFT. Short-time Fourier transform (STFT), wavelet, and event-related spectral perturbation (ERSP) have been frequently applied (Fig. 4b). For example, using these methods, Krokos and Varshney (2022) was able to find a correlation between continuously recorded subjective discomfort (i.e., sickness level from joystick responses) and spectral changes during the VR experience.

Considering that most cybersickness-related EEG studies focused on frequency or time-frequency analysis, we organized previous results showing significant differences in the band powers as the level of discomfort increased. Figure 5 indicates the power changes in each frequency band when participants reported severe cybersickness compared to the baseline condition (i.e., lower level of cybersickness). For the delta band, spectral density increased when cybersickness was getting worse (Chang et al. 2013; Choi et al. 2009; Kim et al. 2005). Theta and alpha bands showed inconsistent results. In particular, while several studies showed enhanced alpha power in the higher cybersickness condition (Ahn et al. 2020; Oh and Whangbo 2018), other experiments indicated the opposite result (Celikcan 2019; Chang et al. 2013; Choi et al. 2009). While beta frequency band tended to show a negative correlation between their power and the level of discomfort (Chang et al. 2013; Choi et al. 2009; Heo and Yoon 2020; Kim et al. 2005), gamma band indicated a positive correlation (Lin et al. 2018; Oh and Whangbo 2018). However, it is highly recommended to perform a meta-analysis to clarify the relationship between cybersickness and spectral changes based on statistical evidence.

A few studies focused on the temporal aspect of EEG during cybersickness using an ERP analysis. Averaging neural signals according to time-locked events can induce either negative or positive voltage deflections, which are associated with ERP components (Luck 2005). The name of the component provides its characteristics. For example, the N2 component refers to the second negatively polarized EEG waveform. Ahn et al. (2020) showed that participants who reported a higher level of discomfort exhibited an enhanced P3 component. Wu et al. (2020) attempted to measure cognitive and attentional changes due to cybersickness using a two-choice oddball task. Participants performed the task before and after the VR experience while EEG data was being recorded. The result showed that cybersickness induced reduced response inhibition, indicating increased N2 and decreased P3 components in the deviant condition. Wei et al. (2019) also showed excessive N2 amplitudes in participants with higher susceptibility to motion sickness when they watched coherent rotating dot patterns.

According to Kim et al. (2019a), the accuracy of cybersickness detection using EEG features can be improved when a specific frequency range is targeted. The authors observed a higher detection accuracy when a narrow kernel shape was applied rather than considering a broader range of power changes. In addition, EEG features extracted from the latter part of the VR experience showed higher detection performance (Khoirunnisaa et al. 2018; Kim et al. 2019a; Ko et al. 2013). This result might come from the fact that a longer duration of VR can cause more severe discomfort (Chang et al. 2020; Rebenitsch and Owen 2016). Since it is likely to experience a higher level of cybersickness during the end part of the content, brain activity during this period can serve as a better indicator for cybersickness-related EEG. Taken together, it is recommended to consider both temporal and spectral aspects of EEG to increase the accuracy of cybersickness detection.

**Table 3:** Summary of feature extraction process for included studies

Reference	Feature extraction	Target frequency bands/ component	Absolute or Relative power	Results	FS	C
Ahn et al. (2020)	Frequency(FFT)	Theta (4–7 Hz) Low-alpha (8–9 Hz) High-alpha (10–12 Hz)	Absolute	Alpha (+) in the higher cybersickness group		
	Other methods (connectivity analysis)	Alpha	Absolute	Increased connectivity in the higher cybersickness group		
	Other methods (ERP)	–	–	Increased P3 amplitudes in the higher cybersickness group for motion perception of accelerative speed		
Celikcan (2019)	Frequency (FFT)	Delta (0.1–3 Hz) Theta (4–7 Hz) Alpha (8–14 Hz) Beta (15–30 Hz)	Absolute	Alpha (−)		
Chang et al. (2013)	Frequency (FFT)	Delta (1.5$-$3.5 Hz) Theta (4–7 Hz) Alpha (8–12 Hz) Beta (12.5–25 Hz)	Relative	Delta (+) Theta (+) Alpha (−) Beta (−)		
Chen et al. (2010)	Time-frequency (STFT)	NM	Relative	Alpha (+)		
Choi et al. (2009)	Frequency (FFT)	Delta (1–4 Hz) Theta (4–8 Hz) Alpha (8–13 Hz) Beta (13–30Hz)	Relative	Delta (+) Theta (−) Alpha (−) Beta (−)		
Dennison et al. (2019)	Frequency (FFT)	Delta Theta Alpha Beta Gamma	Absolute	All frequency bands	$\checkmark$	$\checkmark$
Heo and Yoon (2020)	Frequency (FFT)	Delta (1–4 Hz) Theta (4–8 Hz) Alpha (8–13 Hz) Beta (13–30 Hz)	Both	Absolute Theta (+) Relative beta (−)		
Jeong et al. (2018)	Frequency (FFT)	Theta (4–8 Hz) Alpha (8–12 Hz) Low-beta (12–16 Hz) High-beta (16–25 Hz) Gamma (25–45 Hz)	NM	NM		$\checkmark$
Jeong et al. (2019)	Frequency (FFT)	Theta (4–8 Hz) Alpha (8–12 Hz) Low-beta (12–16 Hz) High-beta (16–25 Hz) Gamma (25–45 Hz)	NM	NM		$\checkmark$
Khoirunnisaa et al. (2018)	Time-frequency (wavelet)	Theta (4–8 Hz) Alpha (8–16 Hz) Beta (16–32 Hz)	Relative	Beta	$\checkmark$	$\checkmark$
Kim et al. (2005)	Frequency (FFT)	Delta (0.2–4 Hz) Theta (4–8 Hz) Alpha (8–13 Hz) Slow-alpha (8–10 Hz) Fast-alpha (10–13 Hz) Beta (13–30 Hz) Slow-beta (13–20 Hz) Fast-beta (20–30 Hz) Gamma (30–50 Hz)	Relative	Delta (+) Beta (−)		
Kim et al. (2008)	Frequency (FFT)	Delta (0.2–4 Hz) Theta (4–8 Hz) Alpha (8–13 Hz) Slow-alpha (8–10 Hz) Fast-alpha (10–13 Hz) Beta (13–30 Hz) Slow-beta (13–20 Hz) Fast-beta (20–30 Hz) Gamma (30–50 Hz)	Relative	All frequency bands	$\checkmark$	$\checkmark$
Kim et al. (2019a)	Time-frequency (STFT)	NM	Relative	All frequency bands		$\checkmark$
Kim et al. (2019b)	Frequency (FFT)	Delta (1–4 Hz) Theta (4–8 Hz) Alpha1 (8–10 Hz) Alpha2 (10–12 Hz) Beta1 (12–18 Hz) Beta2 (18–30 Hz) Gamma (30–50 Hz)	Absolute	Alpha (+)		
	Other methods (source localization)	–	–	In the alpha2 band, posterior cingulate gyrus (PCG) regional source activity was significantly associated SSQ.		
Ko et al. (2013)	Time-frequency (STFT)	Delta (0.1–3 Hz) Theta (4–7 Hz) Alpha (8–13 Hz) Beta (13–20 Hz) Gamma (21–50 Hz)	Absolute	Beta Gamma	$\checkmark$	$\checkmark$
Krokos and Varshney (2022)	Time-frequency (ERSP)	Delta (1–4 Hz) Theta (4–7 Hz) Alpha (7–13 Hz) Beta (13–25 Hz)	Relative	Delta (+) Theta (+) Alpha (+)		
Lee et al. (2019a)	Time-frequency (STFT)	NM	NM	NM		$\checkmark$
Lee and Alamaniotis (2020)	Other methods (binary label)	NM	Absolute	NM		$\checkmark$
Lee et al. (2021)	Frequency (FFT)	Delta (0.5–4 Hz) Theta (4–8 Hz) Alpha (8–13 Hz) Beta (13–30 Hz)	Relative	Delta Theta	$\checkmark$	
Li et al. (2019)	Frequency (FFT)	Theta (4–8 Hz) Alpha (8–13 Hz)	Relative	All frequency bands	$\checkmark$	$\checkmark$
Li et al. (2020)	Time-frequency (wavelet)	Delta (0.5–3 Hz) Theta (4–7 Hz) Alpha (8–13 Hz) Beta (14–30 Hz)	Relative	All frequency bands		$\checkmark$
Liao et al. (2020)	Frequency (FFT)	Delta Theta Low-alpha High-alpha Low-beta High-beta Low-gamma High-gamma	Relative	All frequency bands		$\checkmark$
Lin et al. (2007)	Time-frequency (ERSP)	NM	Relative	Alpha (+)		
Lin et al. (2018)	Frequency (FFT)	Delta (1–4Hz) Theta (4–8Hz) Alpha (8–12Hz) beta (12–25Hz) Gamma (25–50Hz)	Relative	Gamma (+)		
Liu et al. (2020)	Frequency (FFT)	Delta (0–4 Hz Theta (4–8 Hz) Alpha (8–12 Hz) Beta (12–30 Hz) Gamma (30–50 Hz)	Relative	Alpha (-)		
	Other methods (Kolmogorov Complexity)	–	–	Significant decrease in KC with the onset of cybersickness		
Mawalid et al. (2018)	Frequency (FFT)	Alpha (8–13 Hz) Beta (13–20 Hz)	Relative	Alpha Beta		$\checkmark$
Naqvi et al. (2014)	Frequency (FFT)	Alpha	Relative	Alpha (−)		
Naqvi et al. (2015)	Frequency (FFT)	Delta (1.0$-$3.5 Hz) Theta (4$-$7.5 Hz) Alpha (8–12 Hz) Beta (12.5–25 Hz) High-beta (25.5–30 Hz)	Both	Absolute theta (−) Relative beta (−)		
Oh and Whangbo (2018)	Frequency (FFT)	Delta Theta Low-alpha High-alpha Low-beta High-beta Low-gamma Mid-gamma	Relative	Theta (+) Alpha (+) Beta (+) Gamma (+)		
Pane et al. (2018)	Time-frequency (wavelet)	Theta (4–8 Hz) Alpha (8–12 Hz) Beta (12–30 Hz)	Relative	Theta Beta		$\checkmark$
Wei et al. (2011)	Time-frequency (STFT)	1–50 Hz	Relative	All frequency bands	$\checkmark$	$\checkmark$
Wei et al. (2019)	Other methods (ERP)	–	–	Increased N223 in the higher cybersickness group		
	Other methods (phase synchronization)	Theta (4–7 Hz)	NM	Impaired Theta-band phase synchronization networks in the higher cybersickness group		
Wu et al. (2020)	Other methods (ERP)	–	–	Increased N2 amplitude, decreased P3 amplitude, and delayed P3 latency during a two-choice oddball task in the higher cybersickness group		

The "Results" column indicates major findings in a higher cybersickness condition compared to the baseline. Note that we only insert the name of the frequency band if the author of prior work mentioned a link between cybersickness and a specific band but no directional changes. We put a check mark if a prior work conducted further analyses after the feature extraction

*FS * feature selection, * C* classification

#### Feature selection

The results from feature extraction can be refined with higher relevant information through the feature selection process. According to the survey, 7 studies conducted a feature selection process (Fig. 2). The selected features can include a promising neural correlate of cybersickness or be used as a dataset for classification. Table 4 indicates the selection methods adopted in each study, the methods showing the highest accuracy, the number of extracted features, and detailed results of selected features.

Various approaches such as principal component analysis (PCA), information gain (IG), and correlation-based feature selection (CFS) were used for the feature selection. Several studies have tested different selection methods to narrow down the EEG features highly related to the subjective level of cybersickness (Khoirunnisaa et al. 2018; Wei et al. 2011). The results showed that spectral changes in the frontal lobe were closely related to cybersickness. The power change in the beta band, irrespective of the scalp region, was frequently selected as one of the final EEG features (Table 4, 4th column).

**Table 4:** Summary of feature selection process for included studies

References	Methods	# of selected /total features	Details of selected features
Dennison et al. (2019)	SFFS	13/80	Left frontal: alpha Left motor: theta Left parietal: beta Left occipital: delta, theta, alpha Right frontal: theta, gamma Right motor: delta, theta Right parietal: beta, delta Right occipital: gamma
Khoirunnisaa et al. (2018)	IG, CFS	IG: 5/14 CFS: 3/14	F3, O1, O2: beta
Kim et al. (2008)	PCA	31/45	Fz: alpha, beta Cz: beta, gamma, theta, delta Pz: beta, theta, delta O1: alpha, beta, theta, delta O2: alpha, beta, theta, delta
Ko et al. (2013)	e-IBCGA	–	Parietal: beta, gamma
Lee et al. (2021)	ANOVA	4/32	Fp1: delta, theta Fp2: delta, theta
Li et al. (2019)	PCA	–	–
Wei et al. (2011)	BS, GA	BS: 40/50 GA: 34/50	–

*ANOVA* analysis of variance, *BS* backward selection, *CFS* correlation-based on feature selection, *e-IBCGA* extended inheritable bi-objective combinatorial genetic algorithm, *GA* genetic algorithm, *IG* information gain, *PCA* principal component analysis, *SFFS* sequential forward feature selection

#### Classification

After the feature extraction (or selection), several studies have attempted to predict whether participants experience cybersickness or not based on a classification model. About 40% (15/38) of selected studies applied classification methods. Classification models try to predict the user’s discomfort using cybersickness-related EEG features. These are named as a single modality system if only EEG features are used for the classification, while a multi-modality system considers more than just EEG features such as ECG, body sway, and even content features.

**Fig. 6:**
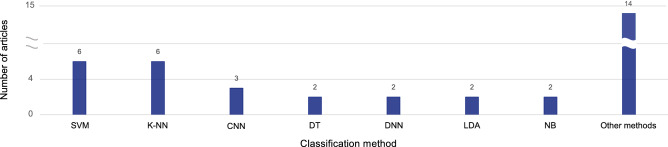
Distributions of suggested approaches in classification. *SVM*: support vector machine, *K*-NN: *k*-nearest neighbors,* CNN* convolutional neural networks,* DT* decision tree,* DNN* deep neural networks,* LDA* linear discriminant analysis,* NB* naive Bayes

Table 5 shows the classifier methods used in the surveyed studies, the highest accuracy of the classifier, and the corresponding method. Most studies adopted two or more classifiers simultaneously and compared the accuracy of each technique. We plotted a bar graph indicating the frequencies of the suggested classifier and found that the support vector machine (SVM) (including the SVM library) and* k*-nearest neighbors (KNN) were the most widely used approaches (Fig. 6). In addition, convolutional neural networks (CNN), decision tree (DT), and deep neural network (DNN) were also frequently applied to predict cybersickness.

The accuracy can be computed based on how accurately the algorithm can predict the subjective level of either an individual’s discomfort (single-subject prediction) or considering all participants on average (multi-subject prediction). The results indicate that using a classifier based on EEG features can identify cybersickness symptoms with a minimum accuracy of 79%. Higher accuracy can also be found in single-subject prediction compared to multi-subject (Table 5, 3rd column).

**Table 5:** Summary of classification process for included studies

References	Methods	Highest accuracy (single-modality)		Note
		Single-subject	Multiple-subject	
Dennison et al. (2019)	ADAB2, **Bag**, DT,* K*-NN, LDA, NB	–	93.8$\%$	95.0$\%$ for multiple-subject prediction using a multi-modality classifier (EEG + ECG, RSP, EGG, body sway)
Jeong et al. (2018)	**DNN**	99.1$\%$	98.5$\%$	
Jeong et al. (2019)	CNN, **DNN**	98.0$\%$	94.3$\%$	
Khoirunnisaa et al. (2018)	*K*-NN, **LDA**, RBF-SVM	–	100$\%$	
Kim et al. (2008)	**ANN**	–	80.0$\%$	
Kim et al. (2019a)	**CNN**, RNN	87.1$\%$	–	89.2$\%$ for single-subject prediction using a multi-modality classifier (EEG + content features)
Ko et al. (2013)	e-IBCGA, **SVM**	97.0$\%$	–	
Lee et al. (2019a)	**Physiological fusion net**	–	–	0.830 (Pearson coefficient) for single-subject prediction using a multi-modality classifier (EEG + ECG, GSR, content features)
Lee and Alamaniotis (2020)	**DESOM**,* K*-NN, SOM	–	0.97 $*$	$*$ Purity index
Li et al. (2019)	*K*-NN, LR, MPNN, RF, **Voting classifier**	–	–	91.1$\%$ for single-subject prediction using a multiple-modality classifier (EEG + body sway)
Li et al. (2020)	*K*-NN, **polynomial-SVM**, RBF-SVM	92.9$\%$	79.3$\%$	
Liao et al. (2020)	MLP, LibSVM, CNN, **LSTM**	–	82.8$\%$	
Mawalid et al. (2018)	*K*-NN, **NB**	–	83.8$\%$	
Pane et al. (2018)	**CN2 Rules**, DT, SVM	–	88.9$\%$	
Wei et al. (2011)	**RBFNN**	84.4$\%$	–	

*Purity index

The classifier in bold showed the best accuracy

*ADAB2* ADABoostM2, *ANN* artificial neural network, *Bag* bagged decision tree, *CNN* convolutional neural network, *DT* decision tree, *DESOM* deep embedded self-organizing map, *DNN* deep neural network, *ECG* electrocardiogram, *EEG* electroencephalogram, *EGG* electrogastrogram, *e-IBCGA* extended inheritable bi-objective combinatorial genetic algorithm, *GSR* galvanic skin response, *K-NN* *k*-nearest neighbor, *LDA* linear discriminant analysis, *LR* logistic regression, *LSTM* long short term memory, *MLP* multilayer perceptron, *MPNN* multilayer perceptron neural network, *NB* naive Bayes, *RBFNN* radial basis function neural network, *RBF-SVM* radial basis function SVM, *RF* random forest, *RNN* recurrent neural network, *RSP* respiration, *SOM* self-organizing map, *SVM* support vector machine

Besides EEG features, other physiological signals or visual features of VR content were included in the classifier. For example, Dennison et al. (2019) developed a multi-modality classifier adding ECG, RSP, EGG, and body sway responses. The accuracy of the multimodal system was 95.0%, which was slightly higher than a single-modality classifier (93.8%). Kim et al. (2019a) extracted visual features from 44 VR content clips and combined these features with a CNN-based EEG classifier. The suggested method, named CNN-RNN network, achieved an accuracy of 89.2% for predicting users’ cybersickness. Lee et al. (2019a) took into account both brain waves and exceptional motion features of visual stimulus to classify the user discomfort. The Pearson linear correlation coefficient (PLCC) was 0.830 when the multimodal system predicted the mean SSQ score.

**Fig. 7:**
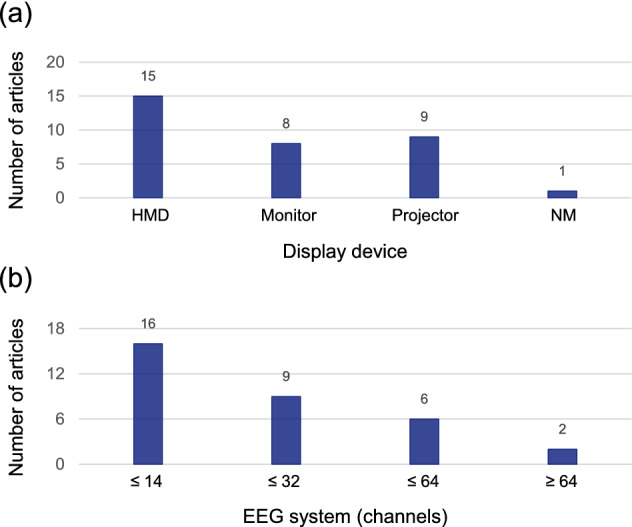
Distributions of hardware setup in** a** display device and** b** EEG system

### Hardware

We investigated the specifications of the hardware systems used in previous studies. For the display devices, most participants experienced VR content using a head mounted display (HMD) (Fig. 7a). Monitors and projectors were also commonly used for presenting the virtual environment. Among these display devices, there can be an issue with electromagnetic (EM) noise on EEG signals when wearing an HMD. Participants usually adjust a strap to firmly attach the HMD device. This movement can influence the contact between the EEG electrode and the scalp. Also, cables from an HMD can move during the experience, which can also cause higher EM noise.

A recent study by Weber et al. (2021) tried to investigate this issue. The results showed that HMDs could induce two types of consistent noise: 50 Hz of line noise and 90 Hz of HMD refresh rate. Fortunately, the typical target frequency of cybersickness-related EEG research is below 50 Hz. Thus, the quality of EEG recordings will remain largely unaffected unless the target frequency is above 50 Hz.

Prior works adopted various ranges of EEG channels, including a single electrode (Liao et al. 2020) to 128 channels (Naqvi et al. 2014, 2015). It is usually recommended to increase the number of electrodes to compensate for EEG’s low spatial resolution. Several studies showed that identifying reliable source dipoles of the brain activity requires more than 32 channels (Michel and Brunet 2019; Srinivasan et al. 1998). However, most of previous studies used less than a 14-channel system (Fig. 7b). This trend might be originated from the context of VR experiments, which already attach many devices to a participant. Using a mobile EEG might help to minimize hardware-originated discomfort. Moreover, using a lower number of EEG channels might due to the hardware being less cumbersome and more affordable for real-world applications.

**Table 6:** Summary of experimental setups for included studies

Article	Display device	# of EEG channels	Content type	Interaction type (sensory feedback)	Content duration	# of participants (analyzed/ recruited)	Age	Subjective measures
Ahn et al. (2020)	HMD (Oculus VR2)	32-channel (BrainAmp DC amplifier)	Scenic (driving)	Passive (low)	6 sec	17/20	M: 29.7 Range: 21–48	SSQ
Celikcan (2019)	HMD (HTC Vive)	14-channel (Emotive Epoc+)	Scenic (roller coaster)	Passive (low)	NM	4/4	M: 28.4 SD: 4.34	SSQ
Chang et al. (2013)	Projector (NM)	64-channel (NeuroScan)	Scenic (roller coaster)	Passive (low)	10 min	20/22	M: 23.91	SSQ
Chen et al. (2010)	Projector (NM)	32-channel (NM)	Scenic (driving)	Passive (Mid/motion bed)	40 min	19/24	M: 22.1	Joystick
Choi et al. (2009)	Projector (NEC MT- I030+ LCD)	2-channel (Biopac MP100)	Scenic (driving)	Passive (low)	60 min	20/20	M: 23.4 SD: 1.8	SSQ
Dennison et al. (2019)	HMD (Oculus Rift DK2)	64-channel (ANT Neuro)	Scenic (navigating)	Active (high)	10 min	20/20	over the age of 18	Nausea scale
Heo and Yoon (2020)	HMD (Baofeng Mojing 3 Plus)	40-channel (Neuroscan Nuamps)	Gaming (fantasy VR)	Active (high)	7.7 min	17/28	M: 26.4 SD: 2.1	SSQ
Jeong et al. (2018)	NM	14-channel (Emotiv Epoc+)	NM (VR videos)	NM	2–3 min	NM/11	NM	NM
Jeong et al. (2019)	HMD (FOVE VR)	14-channel (Emotiv Epoc+)	NM (VR videos)	NM	1–5 min	24/25	Range: 20–33	Keyboard
Khoirunnisaa et al. (2018)	Monitor (47-inch LED)	14-channel (Emotiv Epoc+)	Gaming (mirror edge)	Active (high)	17 min	9/9	M: 25.11	SSQ
Kim et al. (2005)	Projector (theater-type concave screen)	9-channel (Biopac EEG100)	Scenic (navigating)	Active (high)	9.5 min	57/61	M: 23.08; SD: 2.05	SSQ Verbal report Malaise scale
Kim et al. (2008)	Projector (theater-type concave screen)	9-channel (Biopac EEG100)	Scenic (navigating)	Active (high)	9.5 min	43/47	M: 21.23 SD: 2.96 Range: 18–30	SSQ Malaise scale
Kim et al. (2019a)	HMD (HTC Vive)	8-channel (NM)	Scenic (ETRI-VR)	Passive (low)	30 sec	NM/202	NM	Subjective evaluation
Kim et al. (2019b)	HMD (Samsung New Gear VR)	62-channel (NeuroScan SynAmps)	Scenic (VR video clip from YouTube)	Passive (low)	8 min 54 sec	NM/30	M: 25 SD: 4	SSQ
Ko et al. (2013)	Projector (NM)	32-channel (Neuroscan Nuamps)	Scenic (driving)	Passive (Mid/motion bed)	40 min	6/6	NM	Joystick
Krokos and Varshney (2022)	HMD (HTC Vive)	14-channel (Emotiv Epoc+)	Scenic (A fly-through virtual spaceport)	Passive (low)	61 sec	43/44	M: 27 SD: 8	SSQ Joystick
Lee et al. (2019a)	Monitor (LG 34UC98)	30-channel (Cognionics EEG)	360 videos (360 videos from Blend and Vimeo)	Passive (low)	90 sec	17/20	NM	SSQ
Lee and Alamaniotis (2020)	HMD (HTC Vive)	32-channels (Cognionics EEG)	Scenic (roller coaster)	Passive (low)	15 min	19/31	M: 24.04 SD: 2.75	SSQ Mouse
Lee et al. (2021)	HMD (Oculus Rift)	8-channel (BR8 PLUS)	Gaming (Minecraft)	Active (high)	60 min	8/8	M: 21.25	VFQ
Li et al. (2019)	Projector (EPSON)	64-channel (NeuroScan SynAmps2)	Scenic (Visual streaming and car driving video)	Passive (low)	10 min	20/20	M: 22.8	Keyboard
Li et al. (2020)	HMD (HTC Vive)	8-channel (OpenBCI)	Scenic (Navigating)	Passive (low)	< 30 min	18/24	M: 29.3	Switch
Liao et al. (2020)	HMD (HTC Vive)	Single channel (Neurosky Mindwave Mobile)	Scenic (roller coaster, Space simulator, and Boating experience)	Passive (low)	10 min	130/130	Range: 6–23	NM
Lin et al. (2007)	Projector (NM)	32-channel (Neuroscan Nuamps)	Scenic (Car dynamics)	Passive (Mid/ motion bed)	40 min	9/9	M: 22 Range: 18–26	MSQ
Lin et al. (2018)	HMD (NM)	64-channel (NeuroScan)	360 videos (360 videos)	Passive (low)	10 min	25/25	M: 21.96 SD: 2.27	Keyboard
Liu et al. (2020)	Monitor (42-inch LCD)	4-channel (Muse)	Scenic (driving)	Active (mid/ motion bed)	< 30 min	8/8	Range: 20–40	SSQ VIMSL
Mawalid et al. (2018)	Monitor (NM)	14-channel (Emotiv Epoc+)	Gaming (mirror edge)	Active (high)	16 min	9/9	NM	SSQ
Naqvi et al. (2014)	Monitor (42-inch LCD)	128-channel (Geodesic Sensor Net)	Scenic (specialized rotational scenes)	Passive (low)	10 min	6/6	NM	NM
Naqvi et al. (2015)	Monitor (42-inch LCD)	128-channel (Geodesic Sensor Net)	Scenic (specialized rotational scenes)	Passive (low)	10 min	45/52	NM	SSQ
Oh and Whangbo (2018)	HMD (HTC VIVE PRO)	2-channel (Blaubit)	Scenic (roller coaster)	Passive (low)	3 min 10 sec	NM/10	Range: 20–35	NM
Pane et al. (2018)	Monitor (47-inch LED)	14-channel (Emotiv Epoc+)	Gaming (mirror Edge)	Active (high)	16 min	9/9	Range: 25–35	SSQ
Wei et al. (2011)	Projector (NM)	32-channel (Neuroscan Nuamps)	Scenic (driving)	Passive (Mid/ motion bed)	40 min	6/6	NM	Joystick
Wei et al. (2019)	Monitor (46-inch LCD)	32-channel (Neuroscan Nuamps)	Minimalist (coherent or random movement pattern)	Passive (low)	3 sec	27/27	Group 1 M: 24.5; SD: 1.2 Group 2 M: 24.3; SD: 2.7	SSQ
Wu et al. (2020)	HMD (HTC Vive)	24-channel (mBrainTrain)	Gaming (Navigating)	Active (high)	40 min	17/20	M: 25.8 SD: 1.9	SSQ

*MSQ* motion sickness questionnaire, *NM* not mentioned, *SSQ* simulator sickness questionnaire, *VFQ* visual fatigue questionnaire, *VIMSL* visually induced motion sickness level

### Content

**Fig. 8:**
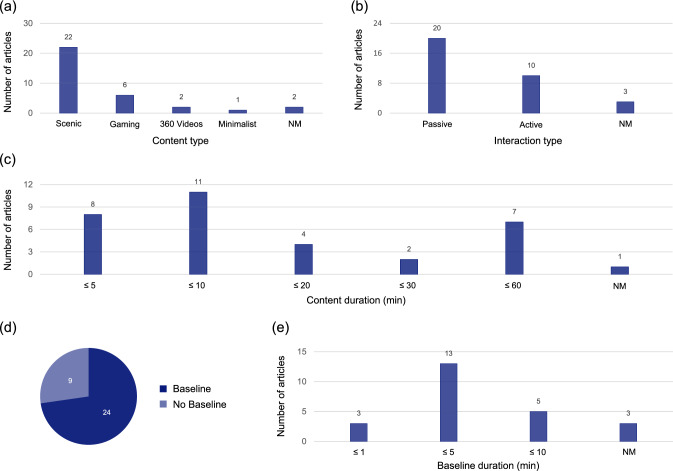
Distributions of ** a** content type,** b** interaction type,** c** content duration,** d** baseline design, and** e** baseline duration

We investigated which VR content was used in the experiments. Based on the content classification from (Saredakis et al. 2020), we organized previously adopted content as scenic, gaming, 360 videos, and minimalist. The result showed that scenic content was the most frequently used in EEG-based cybersickness research (Fig. 8a). In particular, most of the scenic content was a first-person perspective driving simulation in VR with a car, spacecraft, etc. (Table 6, 4th column). Participants were required to drive or navigate in a virtual environment, and the VR scenario contained dynamic and rotational movements to induce a considerable level of cybersickness.

Several commercial gaming applications were also widely used. Compared to the scenic content, gaming VR allowed for active interaction for participants using a controller such as a joystick, mouse, or keyboard (Heo and Yoon 2020; Khoirunnisaa et al. 2018). This interactive experience can also provide a higher level of sensory feedback such as synchronous visual or proprioceptive information (Fig. 8b). A limited number of studies used 360 videos (Lee et al. 2019a; Lin et al. 2018) and minimalist content (Wei et al. 2019).

The length of the contents varied between experiments. Depending on the research hypothesis, participants experienced VR from less than 5 min to an hour (Fig. 8c). For example, in the study of Ahn et al. (2020) and Wei et al. (2019), the visual stimulus was presented for a relatively short period of time (6 s and 3 s, respectively) due to the ERP analysis design. On the other hand, most studies provided sufficient length of VR content to induce a higher level of discomfort. The most widely adopted duration was shorter than 10 min.

Many studies set a baseline session for the experimental design (Supplementary Materials, Appendix 2, Fig. 8d). Using this session, prior works tried to compare the brain activity between the normal (i.e., baseline) and abnormal (VR experience) states. While several studies used simple and slow-moving VR content for the baseline (Chen et al. 2010; Ko et al. 2013), others recorded the resting state (eye-closed or -opened) of a participant (Heo and Yoon 2020; Kim et al. 2019b). The duration of the session was mostly shorter than 5 min (Fig. 8e).

### Other experimental factors

**Fig. 9:**
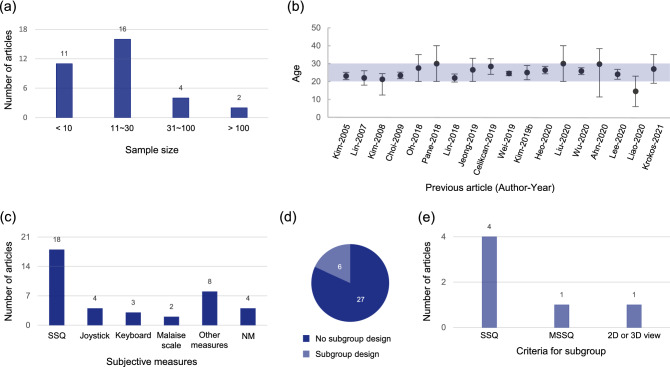
Distributions of the** a** sample size,** b** age,** c** subjective measures for cybersickness,** d** subgroup design, and** e** criteria for subgroup

For other experimental factors, we focused on human-related parts in the prior experiments. First, we investigated the number of participants engaged in the experiments. Most studies recruited less than 30 participants (Fig. 9a), and the dropout rate for EEG analysis was about 6% on average (range 0–39%). Also, it is noted that the age of participants was mostly in their 20 s. Figure 9b illustrates the age distribution of previous studies which reported the mean (dot) and range (line) of their recruited participants.

In agreement with the previous survey Chang et al. (2020), SSQ was the most widely used subjective measure for cybersickness-EEG research Fig. 9c). While the SSQ covers a variety of negative symptoms due to cybersickness, several questionnaires, such as the nausea scale and visual fatigue questionnaire, focus on a specific type of discomfort. Following the SSQ, a joystick or keyboard has also been used to quantify the level of discomfort. Compared to the paper-based measures, these devices made it possible to record participants’ responses in a more continuous and real-time manner (Fig. 6, 9th column).

Several studies divided participants into two groups to investigate the group effect on EEG features (Fig. 9d). One of the criteria that many studies have adopted was the SSQ score (Fig. 9e). That is, a higher SSQ group experienced worse cybersickness compared to a lower SSQ group, and the authors investigated whether this difference was reflected in brain activity. According to the study of Ahn et al. (2020), the higher sickness group showed increased P3 amplitudes when they watched accelerative visual motion stimulation rather than the lower sickness group. Similarly, Choi et al. (2009) found a significant difference in the power spectrum in the frontal and central regions of the brain when comparing the sick and non-sick groups. Also, the difference was enhanced if the participant reported severe discomfort.

Wei et al. (2019) used the score of an individual’s motion sickness susceptibility questionnaire (MSSQ) to identify the group effect on EEG. The results showed that the susceptible group indicated a significant enhancement in the parietal N2 component between two different types of visual patterns compared to the resistant group. The authors suggested that unique cortical activity can be engaged in visual processing depending on one’s susceptibility.

Besides measuring the subjective level of cybersickness, several studies collected individual characteristics which might be associated with discomfort (Supplementary Materials, Appendix 2). For example, Kim et al. (2005, 2008) additionally investigated participants’ motion sickness susceptibility and immersion tendency. Interestingly, only one article collected participant’s previous experience of VR simulation (Lee and Alamaniotis 2020), which is known as a promising human factor for explaining individual difference in cybersickness.

## Discussion

In the current study, we performed a scoping review to provide a preliminary map of cybersickness-related EEG research covering from EEG analyses to the experimental setup. According to our survey, a standardized EEG preprocessing was performed to remove signal noises (i.e., re-reference, resampling, filter, artifact rejection, and epoching) (Fig. 3, Table 2). After the preprocessing, frequency or time-frequency analysis was frequently performed to extract the specific brain activities for cybersickness (Fig. 4, Table 3). Then, several studies conducted feature selection (Fig. 2, Table 4) and/or classification methods (Fig. 6, Table 5) to predict the subjective level of cybersickness using the EEG features. Many studies adopted an HMD and band-type EEG system (less than 14-channel) for the hardware setup (Fig. 7, Table 6). A scenic VR simulation was often used to evoke discomfort (Table 6), and the age of participants was mostly skewed to the 20 s (Fig. 9, Table 6).

For artifact rejection, ICA was the most widely used technique for removing eye movement noise (Bigdely-Shamlo et al. 2015; Kusumandari et al. 2014). In line with this, our survey showed that previous research which reported their artifact rejection method mostly performed ICA analysis. Since VR experiments allow a participant to move one’s eyes voluntarily, there is a higher possibility of contaminating raw EEG signals due to complicated eye movements. For example, Chen et al. (2017) revealed that participants showed decreased alpha power in the smooth pursuit condition (i.e., following a slowly moving target) compared to the fixation condition. In the study of Yuval-Greenberg et al. (2008), involuntarily miniature eye movements (i.e., microsaccade) can induce gamma-band activity. Considering recent studies which showed distinctive eye trajectories during the cybersickness experience (Chang et al. 2021; Wibirama et al. 2020), it is required to clarify whether the EEG spectral changes due to cybersickness are originated from neural oscillations or oculomotor artifacts. According to our survey, about half of the selected studies did not provide a detailed procedure for removing EOG. In future studies, a more thorough explanation of the EOG removal process should be described for a better signal-to-noise ratio of preprocessed EEG.

Most of the previous studies used frequency analysis for feature extraction and tried to clarify the link between a power change in a specific frequency band and the level of cybersickness. Compared to the ERP analysis, this approach can provide a long enough VR experience, to evoke users’ discomfort and is similar to real situations. For example, while previous ERP experiments presented visual stimuli shorter than 10 s (Ahn et al. 2020; Wei et al. 2019; Wu et al. 2020), most frequency analysis experiments provided VR content for more than 5 min to induce a considerable degree of discomfort. Using this approach, researchers have tried identifying which frequency band can be associated with cybersickness. While each frequency band indicated a specific power change due to cybersickness, the alpha band power results were inconsistent between studies (Fig. 5). To reconcile this outcome, we might consider the individual difference in resting state alpha power. Several studies have shown that alpha power asymmetry in the frontal cortex is associated with cognitive control or stress management (Ambrosini and Vallesi 2016; Ma et al. 2021). That is, the inherent difference in a participant’s alpha power can affect the cybersickness-originated brain activity, and it is critical to clarify the source of alpha power changes. Though Kim et al. (2019b) analyzed the resting state EEG to demonstrate the EEG features of cybersickness, they did not take into account individual variability.

Meanwhile, one of the shortcomings of frequency analysis is losing EEG time-domain information, which makes it difficult to explore temporal dynamics during cybersickness. To overcome these shortcomings, recent studies suggested a heartbeat-evoked potential (HEP) which investigates the transient cortical changes according to one’s heartbeats (Chang et al. 2022; Park et al. 2021) while watching continuous VR content. The result showed a significant negative correlation between the HEP amplitude and subjective discomfort, indicating a possible neural index for cybersickness. Further in-depth studies focusing on temporal aspects of EEG are required to detect the level of cybersickness in a real-time manner.

With the rapid progress in machine learning and deep learning techniques, there has been growing interest in using these techniques for detecting cybersickness based on brain signals. Several studies performed feature selection and/or classification, showing the possibility of predicting a participant’s subjective discomfort using EEG features (Dennison et al. 2019; Kim et al. 2008). When classifying multiple subjects’ cybersickness, the accuracy of the classifier was between 79 and 100% (Table 5). Several studies considered the temporal aspects of EEG features to detect cybersickness (Lee et al. 2019a; Liao et al. 2020; Kim et al. 2019a). Kim et al. (2019a) showed 80.57% of test accuracy when considering the longest duration of EEG data. In the study of Liao et al. (2020), the best accuracy was observed in the shortest time step condition (60 sec/ 83.94%), but the difference in accuracy between the longest condition (600 sec/ 83.92%) was 0.02%. Considering the general tendency that longer VR experiences can induce higher cybersickness, using an EEG dataset from the latter part of the content might improve classification accuracy. In addition, as Yildirim (2020) has pointed out, it is important to report a more comprehensive array of classification metrics, availability for data sharing, and detailed information on model design to clarify the classification results.

According to our survey, most experiments adopted HMD-based VR with a band-type EEG system presenting scenic content (driving or navigating simulation). As Weber et al. (2021) mentioned, using HMDs can cause additional electric noise including 50 Hz line noise and 90 Hz refresh rate; therefore, the filter should be carefully designed if the target EEG frequency is higher than 50 Hz. Recently wireless HMDs have been introduced, enabling enjoy various types of VR scenarios (Dianatfar et al. 2023; Gumilar et al. 2022; Lee et al. 2019b). While previous studies have primarily focused on passively experienced content, maintaining a fixed posture for stable EEG signal acquisition, wireless devices can allow dynamic VR experiences such as moving one’s whole body.

Though several studies adopted a motion platform to induce immersive motion feedback (Chen et al. 2010; Ko et al. 2013; Lin et al. 2007; Liu et al. 2020; Wei et al. 2011), these studies have not discussed movement artifacts on brain signals. A recent study by Richer et al. (2020) validated motion and muscle artifact removal using a robotic motion platform. According to the result, the artifacts could be eliminated by recording additional electromyogram (EMG) sensors around the neck. Based on this experimental evidence, it is expected to implement a more interactive VR environment while maintaining high EEG quality if we can attach extra EMG electrodes to a proper location.

In the previous studies, most experiments were performed on undergraduate students in their 20 s due to the easier accessibility of participant recruitment. During the COVID-19 pandemic, the age of VR users is gradually expanding across various context (Ball et al. 2021; Lee and Yoo 2021; Stradford et al. 2021). Despite the increasing interest, little has been known about the age effect on cybersickness-related EEG features. Though several studies indicated a higher incidence of cybersickness in older participants (Arns and Cerney 2005; Knight and Arns 2006), these results were based on subjective measures, which did not take into account physiological signals. According to previous studies (Kaushik et al. 2019; Morgan et al. 2005), the age distribution of participants resulted in a different level of quantitative EEG. For example, Morgan et al. (2005) found a positive correlation between age and beta frequency power. Thus, if the older age group who experience cybersickness shows increased beta power, careful interpretation is required to clarify whether this result originates from aging or cybersickness.

Among several individual characteristics, MSSQ has been known as a promising indicator of the experience of cybersickness. According to Kim et al. (2005), the MSSQ score showed a significant positive correlation with the severity of cybersickness. That is, it is likely to experience severe discomfort if a person is susceptible to motion sickness. In the dataset of the present review, only the study of Wei et al. (2019) investigated the changes in EEG features depending on the MSSQ score. Participants more prone to experience motion sickness (i.e., high MSSQ) showed an increased N2 component and impaired theta-band phase synchronization when they watched a coherent moving pattern. This study used simple visual stimuli (moving dots) with a short duration (3 sec), providing evidence of individual differences in neural processing during low-level motion perception. However, due to the lack of similarity with the real-world VR experience, it is required to perform further experiments to replicate the results. Additional studies using complex VR content for extended periods should be done for future works.

This scoping review has several limitations. First, while we intended to perform a meta-analysis to clarify the EEG spectral correlates of cybersickness, it was not possible to obtain the data of EEG power in each experimental condition (i.e., higher vs. lower cybersickness) from the authors. Except for the study of Kim et al. (2019b), most previous studies only reported statistical differences between two conditions using the *F*- or *t*-test, making it difficult to quantify systematic differences. Future EEG spectral analyses should present the results indicating the mean and SD of each frequency spectrum depending on the discomfort level. Also, due to the limited number of studies, we only selected studies conducted on healthy participants. Arafat et al. (2018) compared the EEG power between multiple sclerosis patients and healthy patients and showed a significant difference in the beta power of the two groups when they were experiencing cybersickness. Considering the growing range of VR applications, more research should be conducted to investigate the cybersickness-related EEG in various populations.

## Conclusion

Among several attempts to quantify cybersickness, EEG has been introduced as one of the promising signals that can objectively measure the level of discomfort. In particular, with the recent growth of the machine learning and deep learning area, there is an increasing interest in detecting and predicting cybersickness based on brain waves. Using the PRISMA-ScR approach, this paper presents a scoping review of cybersickness-related EEG research. Thirty-three articles (38 experiments in total) were selected for the review and surveyed in four categories: EEG analysis pipeline, hardware, content, and other experimental factors. This approach was aimed to investigate whether there are any consistent findings or trends in cybersickness-related EEG research (RQ1). We also tried to investigate the accuracy of the suggested EEG-based classification methods (RQ2) and experimental setups of previous studies (RQ3).

According to our survey, EEG data analysis can be organized into the four steps of preprocessing, feature extraction, feature selection, and classification steps. For the EEG preprocessing, most studies performed a structured pipeline to remove noise from the raw brain signals. After the re-referencing, most studies re-sampled the data between 128 to 256 Hz. Then, the resampled data were filtered using a band-pass filter (mostly 0.1–60 Hz) and EOG artifacts were removed based on the ICA analysis. The epoch of EEG analyses was usually shorter than 5 min. Using the preprocessed data, feature extraction was performed to find the neural correlates of cybersickness. The majority of previous studies conducted frequency or time-frequency analysis, showing increased delta and decreased beta power in a higher cybersickness condition. Some of the works performed feature selection to narrow down the cybersickness-related brain activity. Classification models were then applied to predict the subjective level of discomfort based on the EEG features, achieving more than 79% accuracy. The selected studies frequently used a VR HMD for display and an EEG system with less than 14-channel. The VR experience used to induce cybersickness was most often a navigating or driving scenario, less than 10 min in duration. The typical experiment’s sample size was smaller than 30 participants, and the age range was largely limited to people in their 20 s. For subjective measures, SSQ was the most widely used questionnaire.

The current scoping review contributes to providing a preliminary exploration of the EEG features and experimental setup in cybersickness research. In addition, this research can serve as a guideline for adopting an EEG system in VR research. Based on this discussion, we summarize directions for future cybersickness-related EEG research as follows:

- Investigating the effect of eye movements on EEG to clarify the cybersickness-related EEG features
- Applying EEG analyses that consider temporal aspects of cybersickness-originated brain activity
- Indicating quantitative results in the higher and/or lower cybersickness condition (e.g., relative power (dB), amplitude (μV), etc.) to conduct a meta-analysis
- Providing comprehensive classification metrics, data sharing, and detailed information on model design
- A broader range of VR content that provides active interactions and higher sensory feedback
- Including various populations with a broader age range

## Supplementary Information

Below is the link to the electronic supplementary material.

Supplementary file 1 (pdf 90 KB)

Supplementary file 2 (xlsx 180 KB)

## Data Availability

All data generated or analyzed during this study are included in this published article [and its supplementary information files].
